# Iodide of Zinc as a Local Application

**Published:** 1857-04

**Authors:** 


*Iodide of Zinc as a Local Application.*

In the March number of the *Peninsular Journal*, it is stated that Dr. Pitcher, of Detroit, had recently used a solution of iodide of zinc to the conjunctiva when left thickened and granular after previous attacks of inflammation. We have also been in the habit of occasionally using the same remedy in very chronic cases, and with the most decided benefit. We apply it with a camel-hair pencil, and vary the strength in different cases, from ℈j. to ʒj. to the ounce of water. The application should not be repeated oftener than every second or third day.

Recently, we applied the same remedy (iodide of zinc ℈j. to water ℥j.) to the os and neck of the womb, and the upper part of the vagina, in two very protracted and bad cases of leucorrhœa. The application was made by means of a sponge attached to a piece of whalebone, and introduced through the speculum. It required a repetition of the application only three or four times, at intervals of three days, to suppress the discharge altogether and much improve the feelings of the patients.

